# Transient Cell Membrane Disruptions induce Calcium Waves in Corneal Keratocytes

**DOI:** 10.1038/s41598-020-59570-7

**Published:** 2020-02-18

**Authors:** Zhong Chen, Xiaowen Lu, Meghan E. McGee-Lawrence, Mitchell A. Watsky

**Affiliations:** 1 Department of Cellular Biology and Anatomy, Medical College of Georgia, Augusta University, Augusta, Georgia USA; 2 Department of Orthopedic Surgery, Medical College of Georgia, Augusta University, Augusta, Georgia USA; 3 The Graduate School, Augusta University, Augusta, GA Georgia

**Keywords:** Ion channel signalling, Medical research

## Abstract

The purpose of this study was to determine if transient cell membrane disruptions (TPMDs) in single keratocytes can trigger signaling events in neighboring keratocytes. Stromal cells were cultured from human corneas (HCSC) and mouse corneas (MCSC). TPMDs were produced using a multiphoton microscope in Cal-520-AM loaded cells. TPMD-induced calcium increases (Ca^++^_i_) were measured in Ca^++^-containing and Ca^++^-free solutions containing thapsigargin, ryanodine, BAPTA-AM, 18-α-glycyrrhetinic acid (18α-GA), apyrase, BCTC, AMG 9810, or AMTB. Fluorescence intensity was recorded as the number of cells responding and the area under the fluorescence versus time curve. The maximum distance of responding neighboring cells in *ex vivo* human corneas was measured. Connexin 43 protein in HCSC and MCSC was examined using immunofluorescence staining, and corneal rubbing was applied to confirm whether TPMDs occur following mechanical manipulation. Our results demonstrate that single cell TPMDs result in Ca^++^ waves in neighboring keratocytes both in culture and within *ex vivo* corneas. The source of Ca^++^ is both intra-and extra-cellular, and the signal can be mediated by ATP and/or gap junctions, and is species dependent. Stromal rubbing confirmed that TPMDs do occur following mechanical manipulation. Keratocyte TPMDs and their associated signaling events are likely common occurrences following minor or major corneal trauma.

## Introduction

The cornea is the anterior-most segment of the eye, and is the most powerful refractive element of the eye. Histologically, the cornea contains an outwardly-facing epithelium, a stroma containing keratocytes and nerves, and in inner-facing endothelium. Stromal keratocytes are dispersed among stromal collagen fibers, forming a network of cells interconnected via gap junctions^1–4^. Keratocytes are typically quiescent under normal physiological conditions. Following corneal injury, keratocytes activate and become myofibroblasts, which migrate to the wound area and are heavily involved in the wound repair process.

In many cell types, including gastrointestinal tract cells, myocytes, and osteocytes, physiological mechanical loading can create transient micro-tears in the cell membrane termed transient plasma membrane disruptions (TPMDs)^5–8^, which is a common form of cell injury. These TPMDs are typically repaired rapidly (within 10 to 60 seconds) to allow for continued cell survival. Importantly, TPMDs foster molecular flux across cell membranes and promote tissue adaptation by initiating cellular mechanotransduction signaling. A TPMD facilitates rapid extracellular calcium influx at the site of membrane injury, which can trigger a number of downstream intra- and extracellular signaling events.

TPMDs and their associated intercellular signaling responses may occur frequently in corneal keratocytes as a result of mechanical stimulation initiated by, for example, eye rubbing and minor or major corneal trauma. Mechanical stimulation has been postulated to be the source of TPMDs in bone osteocytes, which reside in bony lacunae and are consequently much more protected from external environmental influences than keratocytes^7^. Recently, one of the authors (MML) introduced the use of the multi-photon microscope laser to induce TPMD in osteocytes^7^, methodology that has been used previously in other cells like myocytes and keratinocytes^9,10^. This study demonstrated single cell TPMD-initiated calcium signaling in both the wounded cell and non-wounded adjacent neighbors, and also found that ATP was important for signaling mechanisms downstream of TPMD. Interestingly, in addition to signaling to neighboring cells, the TPMD-induced influx of extracellular calcium into the wounded osteocyte’s cytosol through the membrane tear was shown to initiate a signaling response leading to the activation of the plasma membrane repair machinery^7,8^. The primary focus of TPMD research up until recently has been examination of how these wounds heal themselves as opposed to the resulting effects on neighboring cells.

Gap junctions and ATP have both been shown to promote intercellular calcium wave propagation^11^. Gap junctions are specialized plasma membrane structures that provide a communication pathway among adjacent cells, facilitating direct exchange of small molecules (<1000 Da) including ions, metabolites and second messengers (e.g., calcium, glucose, cAMP, cGMP, IP3)^12^. Gap junction-mediated communication is important in maintaining corneal homeostasis, and also plays a vital role in mediating corneal wound healing^13–15^. Unlike typical calcium-wave propagation, gap junctions do not appear to be a conduit for bone cell TPMD-induced calcium wave propagation. The current study examined the influence gap junctions on corneal keratocyte TPMD-induced calcium wave propagation.

Activation of plasma membrane calcium channels are a likely source of calcium wave propagation. While many corneal keratocyte signaling pathways have been previously described (e.g. reviewed in^16,17^), the only keratocyte calcium transport pathways described to date are through the TRPV1 and TRPM8 channels^18,19^. The role of these two channels in keratocyte TPMD-induced calcium wave propagation was examined in the current study.

In this study, we demonstrate and describe TPMD-induced calcium signaling within the mouse and human keratocyte networks. We also demonstrate TPMD-induced calcium waves within any cells residing in their native tissue for the first time. In addition, this study demonstrates the relationship between keratocyte TPMD-induced calcium waves, gap junctions, and ATP.

## Results

### Calcium signaling

#### Extracellular calcium

**Figure 1:**
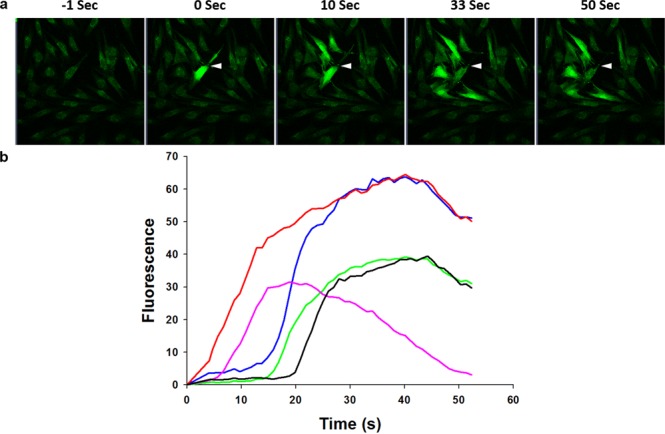
TPMD initiates calcium waves in human corneal stromal cells. (**a**) Representative trial showing cells at different time points following a TPMD. White arrow shows TPMD site; other cells were not wounded. (**b**) Ca^++^ fluorescence intensity versus time curves of five selected cells from top panel (does not include the source cell) demonstrate Ca^++^ signaling magnitude over time following a TPMD.

Creation of a single, small TPMD in single keratocytes/stromal cells of all control preparations resulted in large Ca^++^_i_ increases in the targeted (source) cells and in many neighboring cells in a time-dependent fashion (i.e. Ca^++^ waves; Fig. 1). A summary of all experimental results is presented in Table 1.

**Table 1:** Summary of Experimental Treatment Results.

Treatment	Number		Area		Distance
	H Cells	M Cells	H Cells	M Cells	H Corneal Rim Tissue
K-SFM*	↓	↓	↓	↓	↓
Thapsigargin (1 uM, 1 h)	↓	↓	NS	NS	NS
18α-GA (30 mM, 1 h)	NS	NS	NS	↓	↓
Apyrase (10 U/ml, 30 min)	↓	NS	↓	NS	N/A
BCTC (10 mM, 1 h)	NS	NS	NS	NS	NS
AMG 9810 (10 uM, 30 min)	NS	↓	NS	NS	N/A
AMTB (10 uM, 30 min)	NS	NS	NS	NS	N/A
K-SFM + Thapsigargin (1 uM, 1 h)*	↓	↓	↓	↓	↓
K-SFM + Ryanodine (50 uM, 1 h)*	↓	↑	↓	↓	N/A
K-SFM + BAPTA-AM (50 uM, 1 h)*	↓	↓	↓	↓	N/A

Note: H: human; M: mouse.

↓ reduced; ↑ increased; NS: not significantly different compared to DMEM; N/A: not applicable.

*Compared to K-SFM + 1 mM calcium.

**Figure 2:**
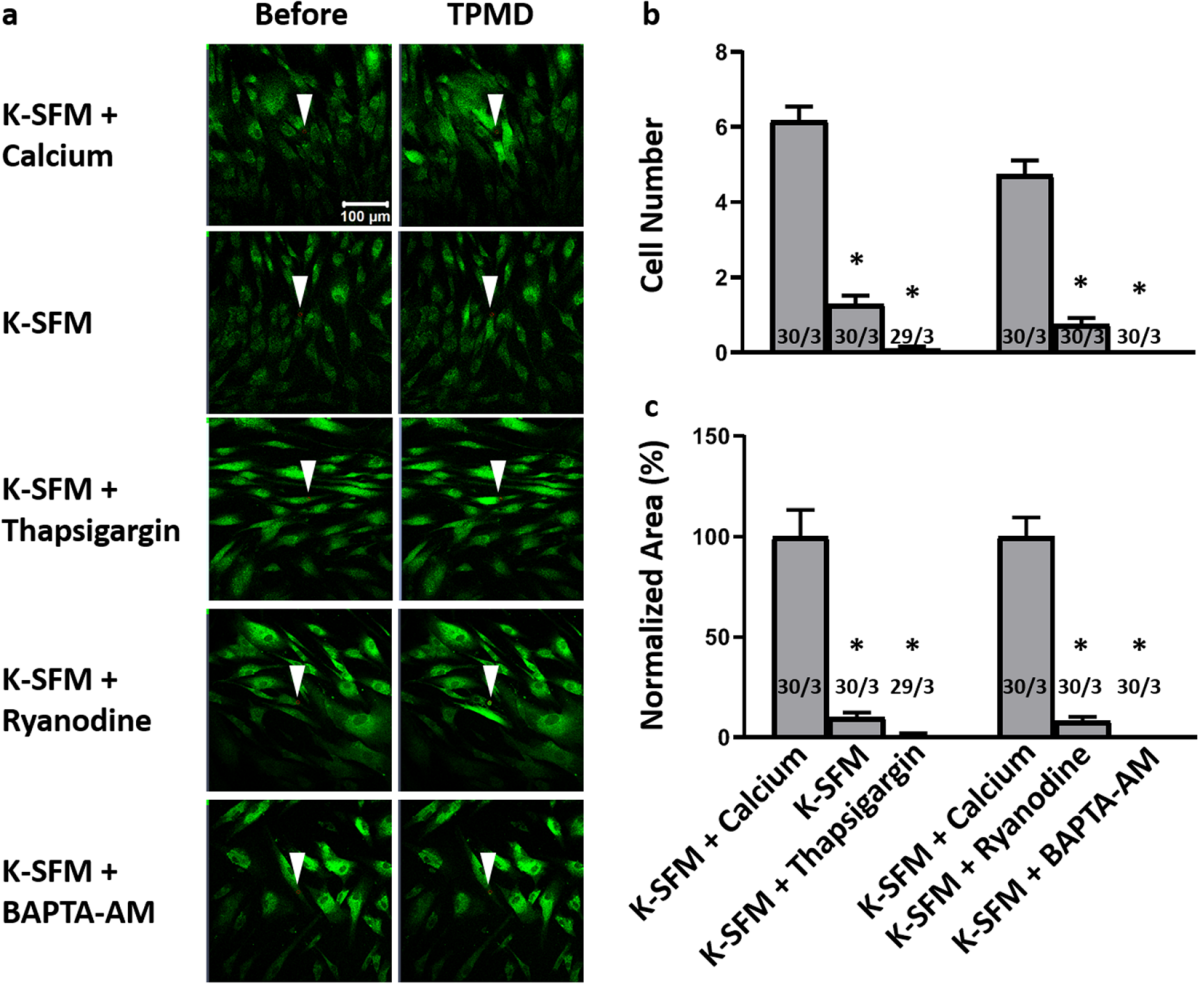
TPMD-induced calcium signaling in primary human corneal stroma cells. (**a**) Representative images of Cal-520-AM stained human stroma cells before and after laser-induced TPMD. The TPMD location is shown as an arrowhead (laser target). K-SFM was used as a Ca^++^-free extracellular medium and K-SFM + 1 mM Ca^++^ was used as a Ca^++^ positive control. (**b**) Number of neighboring cells in the visual field that had at least a 50% increase in fluorescent intensity following source cell TPMD. (**c**) Normalized area under the intensity versus time curve of neighboring cells with at least a 50% increase in fluorescent intensity. Numbers within bars indicate TPMD targeted number of cells/number of plates examined. Data presented as mean ± SE. * indicates P < 0.05.

Initial experiments were designed to determine the Ca^++^ source of neighboring cell TPMD-induced Ca^++^ waves. Cultured corneal stromal cells and keratocytes within cornea rims were examined in K-SFM with or without Ca^++^ to examine the influence of extracellular calcium. Figure 2a shows representative micrographs of HCSC before and after TPMD under several experimental conditions. It is notable that Ca^++^-free K-SFM significantly reduced both the human stromal cell responding number (1.3 ± 0.22) and normalized curve area (9.83% ± 2.56) when compared to K-SFM + 1 mM calcium (6.16 ± 0.38, 100% ± 13.39; both P < 0.05) (Fig. 2b,c). Calcium wave videos corresponding to all of the still photographs in Fig. 2 can be found in Supplemental Videos S1–S5.

**Figure 3:**
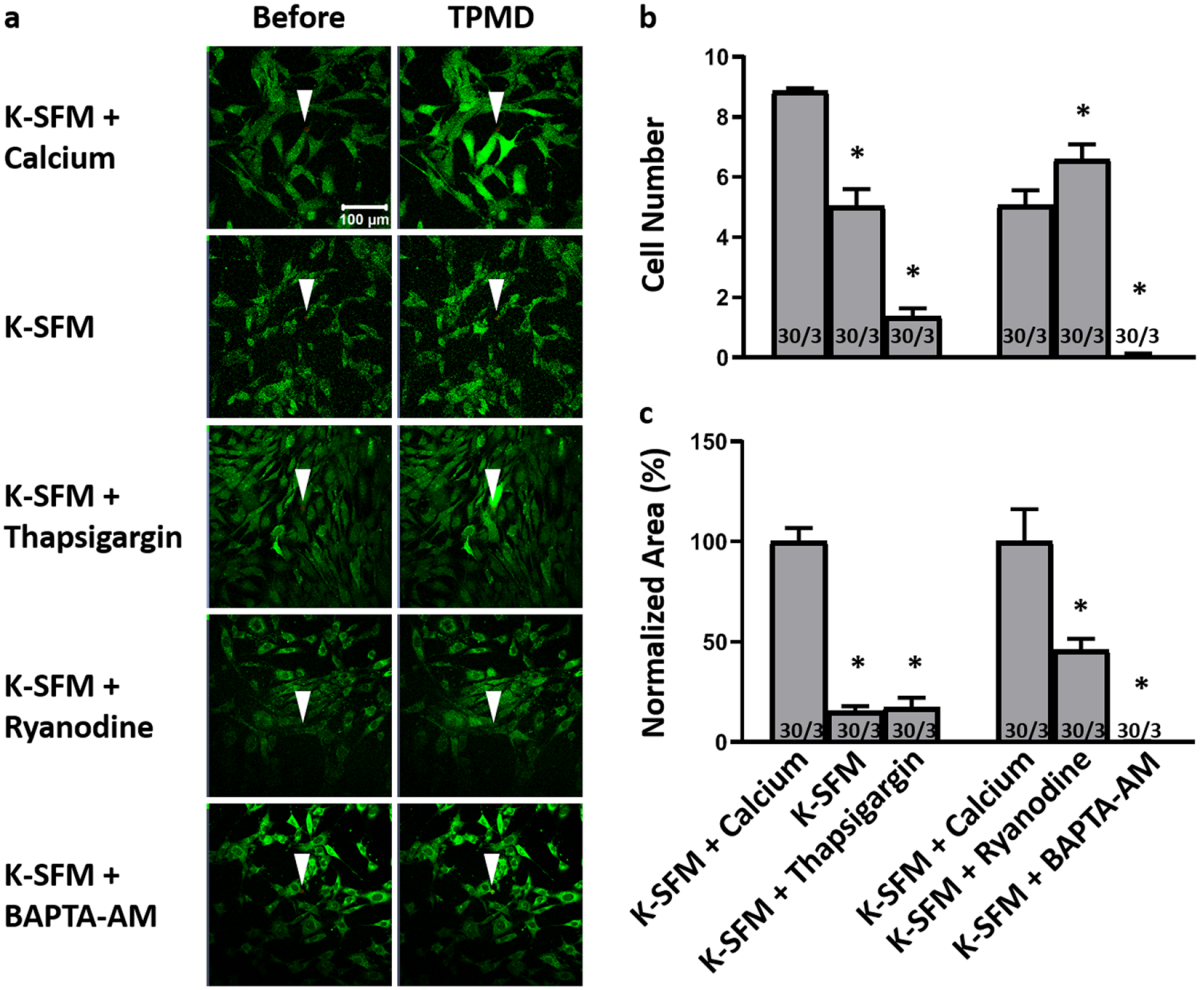
TPMD-induced calcium signaling in primary mouse corneal stroma cells. (**a**) Representative images of Cal-520-AM stained mouse stroma cells before and after laser-induced TPMD. The TPMD location is shown as an arrowhead. K-SFM used as a Ca^++^-free extracellular medium and K-SFM + 1 mM Ca^++^ was used as a Ca^++^ positive control. (**b**) Number of neighboring cells in the visual field that had at least a 50% increase in fluorescent intensity following source cell TPMD. (**c**) Normalized area under the intensity versus time curve of neighboring cells that had at least a 50% increase in fluorescent intensity. Numbers within bars indicate TPMD targeted number of cells/number of plates examined. Data presented as mean ± SE. ^*^ indicates P < 0.05.

Figure 3a demonstrates representative micrographs of MCSC before and after TPMDs. In cultured primary mouse corneal stromal cells, Ca^++^-free K-SFM resulted in a significantly lower cell number (5.03 ± 0.56) and normalized curve area (15.57% ± 2.38) compared to K-SFM + 1 mM calcium (8.86 ± 0.09 and 100% ± 6.75, respectively; P < 0.05) (Fig. 3b,c). Videos corresponding to all of the still photographs in Fig. 3 can be found in Supplemental Videos S6–S10.

**Figure 4:**
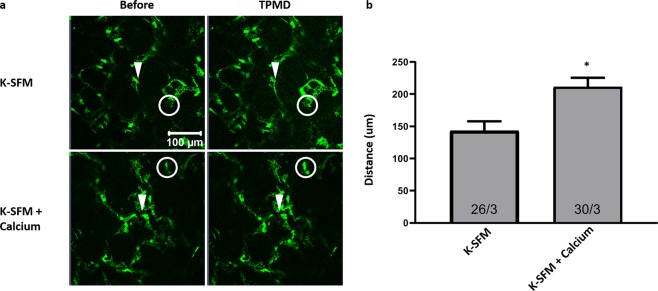
Ca^++^-free K-SFM reduces TPMD-induced keratocyte calcium signaling in *ex vivo* human corneal rims. (**a**) Representative images of Cal-520-AM stained keratocytes within *ex vivo* human corneal rims bathed in Ca^++^-free K-SFM and K-SFM + Ca^++^ before and after laser-induced TPMD. The TPMD location is shown as an arrowhead. The neighboring cell farthest from the source cell with a notable change in fluorescence was noted (white circle) and the distance from the source cell was measured. The mean maximum distance of approximately 10 target source cells from each rim was calculated and used for statistics analysis. (**b**) Ca^++^-free K-SFM versus K-SFM + Ca^++^ cell distance. Numbers within bars indicate TPMD targeted number of cells/number of rim. Data presented as mean ± SE. ^*^ indicates P < 0.05.

The influence of extracellular calcium on TPMD-induced calcium signaling was also studied in keratocytes residing *in situ* within human corneal rim tissue. Our results confirm that TPMD-induced keratocyte calcium signaling is present within corneal tissue (Fig. 4a). As in the cultured cells, calcium signaling was significantly reduced in a Ca^++^-free extracellular environment (Fig. 4a,b). The mean maximum cell distance between the source cell and farthest responding cell was 143.43 ± 14.28 μm in the Ca^++^-free K-SFM group vs. 211.57 ± 13.9 um in the K-SFM + 1 mM calcium group (P < 0.05). Videos corresponding to all of the still photographs in Fig. 4 can be found in Supplemental Videos S11–S12.

#### Intracellular calcium

K-SFM plus the sarcoplasmic/endoplasmic reticulum Ca^++^ ATPase inhibitor thapsigargin, or the intracellular Ca^++^ release blocker ryanodine, were used to examine the role of intracellular Ca^++^ in TPMD-induced calcium waves. K-SFM plus the calcium chelator BAPTA-AM was as a positive control to examine the combined extracellular and intracellular calcium influence on TPMD-induced calcium waves.

In HCSC, K-SFM + thapsigargin significantly reduced both responding cell number (0.10 ± 0.05) and normalized curve area (1.12% ± 0.89) when compared to K-SFM + 1 mM calcium (6.16 ± 0.38, 100% ± 13.39; both P < 0.05) (Fig. 2b,c). K-SFM + ryanodine and K-SFM + BAPTA-AM significantly reduced both the human stromal cell responding number (K-SFM + ryanodine: 0.76 ± 0.15; K-SFM + BAPTA-AM: 0.00 ± 0.00) and normalized curve area (K-SFM + ryanodine: 8.06% ± 2.1; K-SFM + BAPTA-AM: 0.00% ± 0.00) when compared to K-SFM + 1 mM calcium (4.73 ± 0.37 and 100% ± 9.69, respectively; P < 0.05) (Fig. 2b,c).

In MCSC, K-SFM + thapsigargin significantly reduced both responding cell number (1.36 ± 0.27) and normalized curve area (17.38% ± 4.87) when compared to K-SFM + 1 mM calcium (8.86 ± 0.09 and 100% ± 6.75, respectively; P < 0.05) (Fig. 3b,c). K-SFM + BAPTA-AM significantly reduced both cell number (0.06 ± 0.06) and normalized curve area (0.25% ± 0.25) when compared to K-SFM + 1 mM calcium (5.06 ± 0.49, 100% ± 16.17, respectively; P < 0.05). K-SFM + ryanodine also significantly reduced normalized curve area (45.81% ± 5.74, P < 0.05), but interestingly, it increased cell number (6.6 ± 0.48, P < 0.05) when compared to K-SFM + 1 mM calcium (Fig. 3b,c).

**Figure 5:**
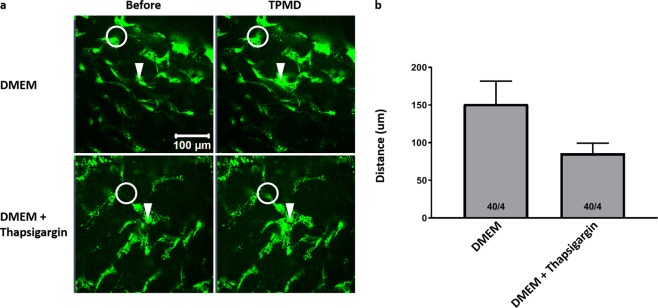
No effect of sarcoplasmic reticulum Ca^++^ ATPase inhibition on TPMD-induced keratocyte calcium signaling in *ex vivo* human corneal rims. (**a**) Representative images of Cal-520-AM stained keratocytes within *ex vivo* human corneal rims bathed in DMEM and DMEM + thapsigargin (1 μM for 1 h) before and after laser-induced TPMD. The TPMD location is shown as an arrowhead. The neighboring cell farthest from the source cell with a notable change in fluorescence was noted (white circle) and the distance from the source cell was measured. The mean maximum distance of approximately 10 target source cells from each rim was calculated and used for statistics analysis. (**b**) DMEM versus DMEM + thapsigargin cell distance. Numbers within bars indicate TPMD targeted number of cells/number of rim. Data presented as mean ± SE.

The influence of intracellular calcium on TPMD-induced calcium signaling was also studied in keratocytes residing within human corneal rim tissue. Thapsigargin did not significantly reduce the cell distance in stromal keratocytes (151.20 ± 30.45 μm in DMEM controls vs. 85.74 ± 13.63 μm in the thapsigargin group) (Fig. 5). Videos corresponding to all of the still photographs in Fig. 5 can be found in Supplemental Videos S13–S14. Thapsigargin added to K-SFM did significantly reduce the cell distance (52.26 ± 1.80 μm in K-SFM + thapsigargin vs 211.58 ± 13.90 μm in K-SFM + 1 mM calcium; P < 0.05).

### Influence of gap junctions

**Figure 6:**
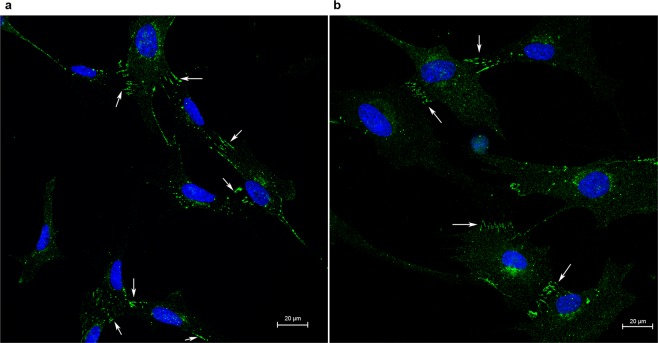
Gap junction Cx43 immunofluorescence staining. Representative immunostain images demonstrate the presence of Cx43 (white arrows) at the cell junctions of primary (**a**) human and (**b**) mouse corneal stromal cells.

To confirm the presence of gap junctions in HCSC and MCSC, Cx43 immunofluorescence staining was performed. Figure 6 demonstrates the presence of Cx43 at the cell junctions of both HCSC and MCSC.

**Figure 7:**
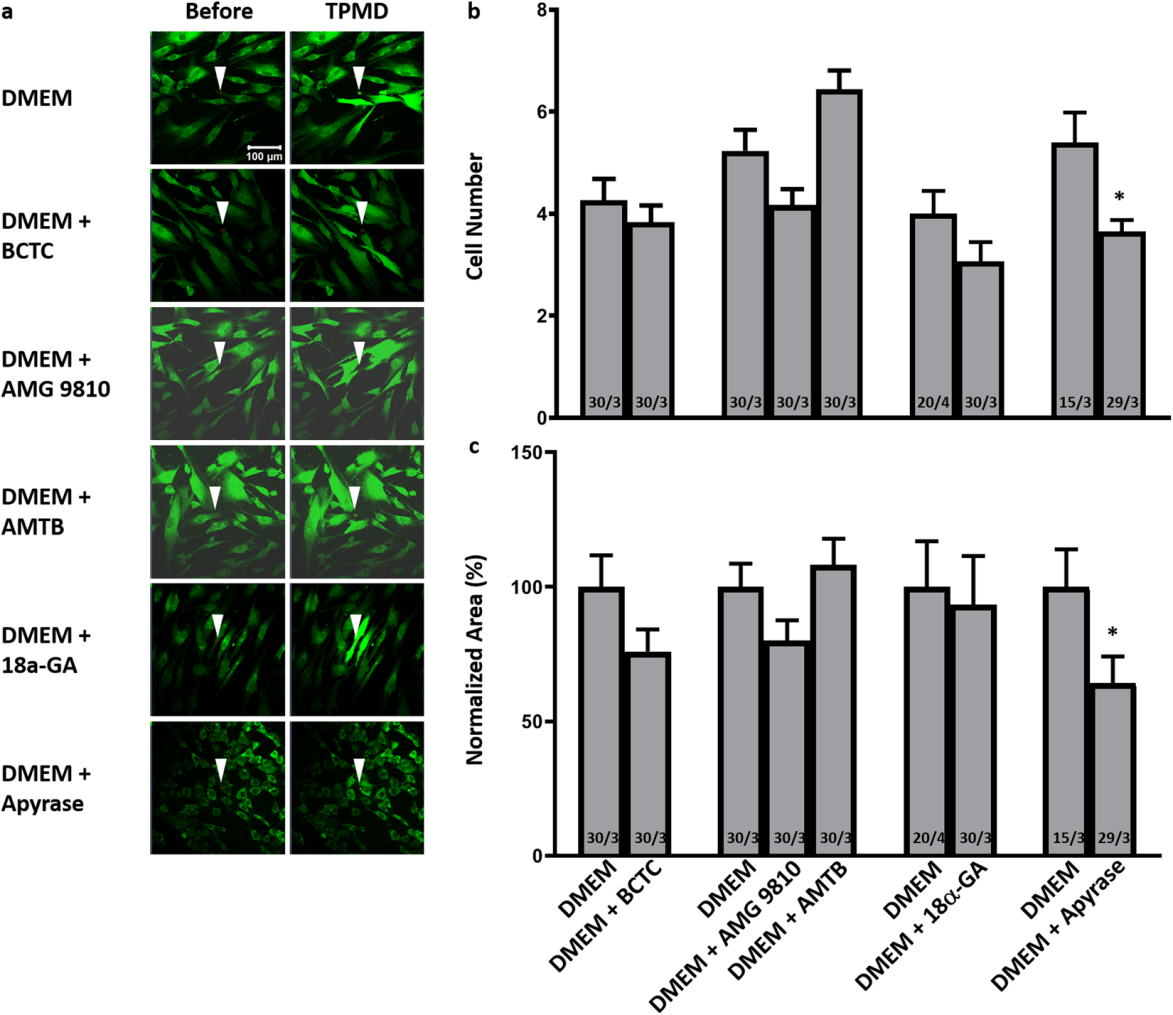
The influence of TRPV1, TRPM8, gap junctions and ATP on TPMD-induced calcium signaling in primary human corneal stromal cells. (**a**) Representative images of Cal-520-AM stained human stroma cells bathed in DMEM and DMEM plus indicated treatments before and after laser-induced TPMD. The TPMD location is shown as an arrowhead. DMEM was used as control. (**b**) Number of neighboring cells in the visual field that had at least a 50% increase in fluorescent intensity following source cell TPMD. (**c**) Normalized area under the intensity versus time curve of neighboring cells that had at least a 50% increase in fluorescent intensity. Numbers within bars indicate TPMD targeted number of cells/number of plates examined. Data presented as mean ± SE. ^*^ indicates P < 0.05.

The influence of gap junctions on TPMD-induced calcium signaling was examined using the gap junction blocker 18α-GA. In primary human corneal stromal cells, there was no significant difference between DMEM controls and 18α-GA treated cells in either the cell number or the normalized curve area (4.00 ± 0.45 vs. 3.07 ± 0.38 and 100% ± 16.99 vs. 93.33% ± 18.13, respectively) (Fig. 7b,c). Videos corresponding to all of the still photographs in Fig. 7 can be found in Supplemental Videos S15–S20.

**Figure 8:**
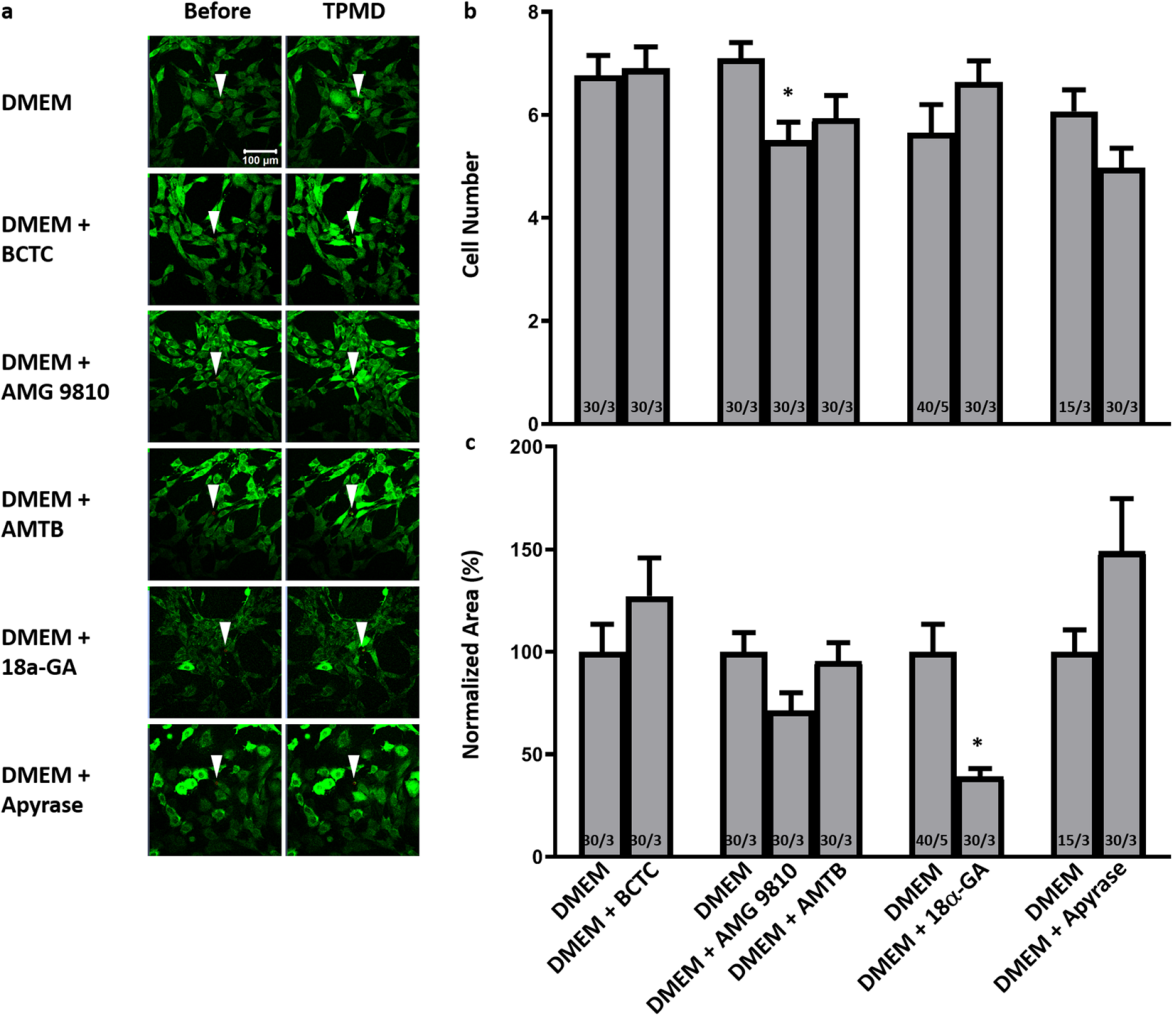
The influence of TRPV1, TRPM8, gap junction and ATP on TPMD-induced calcium signaling in primary mouse corneal stromal cells. (**a**) Representative images of Cal-520-AM stained mouse stroma cells bathed in DMEM and DMEM plus indicated treatments before and after laser-induced TPMD. The TPMD location is shown as an arrowhead. DMEM was used as control. (**b**) Number of neighboring cells in the visual field that had at least a 50% increase in fluorescent intensity following source cell TPMD. (**c**) Normalized area under the intensity versus time curve of neighboring cells that had at least a 50% increase in fluorescent intensity. Numbers within bars indicate TPMD targeted number of cells/number of plates examined. Data presented as mean ± SE. ^*^ indicates P < 0.05.

**Figure 9:**
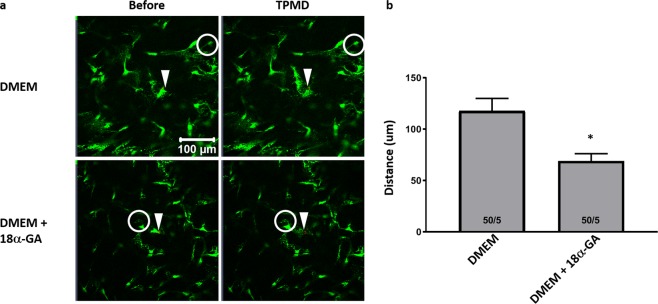
Blocking gap junctions reduces TPMD induced calcium signaling in *ex vivo* human corneal rims. (**a**) Representative images of Cal-520-AM stained keratocytes within *ex vivo* human corneal rims bathed in DMEM and DMEM + 18α-GA (30 μM for 1 h) before and after laser-induced TPMD. The TPMD location is shown as an arrowhead. The neighboring cell farthest from the source cell with a notable change in fluorescence within the visual field was noted (white circle) and the distance from the source cell was measured. The mean maximum distance of approximately 10 target source cells from each rim was calculated and used for statistics analysis. (**b**) DMEM versus DMEM + 18α-GA cell distance. Numbers within bars indicate TPMD targeted number of cells/number of rim. Data presented as mean ± SE. ^*^ indicates P < 0.05.

In primary mouse corneal stromal cells, there was also no significant difference in the cell number between DMEM controls and 18α-GA treated cells (5.65 ± 0.55 versus 6.63 ± 0.42, respectively). There was, however, a significant decrease in the normalized curve area of control versus 18α-GA treated cells (100% ± 13.48 vs 38.96% ± 4.06; P < 0.05) (Fig. 8b,c). Videos corresponding to all of the still photographs in Fig. 8 can be found in Supplemental Videos S21–S26. In human corneal rim tissue compared to DMEM, 18α-GA significantly reduced the cell distance (117.71 ± 12.13 μm vs. 68.92 ± 7.22 μm, respectively; P < 0.05) (Fig. 9). Videos corresponding to all of the still photographs in Fig. 9 can be found in Supplemental Videos S27,S28.

### Influence of extracellular ATP

The influence of extracellular ATP, presumably released by the source cell, on TPMD signaling was analyzed using apyrase, a potent hydrolyzer of ATP. In primary human corneal stromal cells, both the cell number and the normalized curve area were significantly reduced following apyrase treatment (5.40 ± 0.58 vs. 3.66 ± 0.22 and 100% ± 13.94 vs. 64.04% ± 10.06, respectively; P < 0.05) (Fig. 7b,c). In primary mouse corneal stromal cells, apyrase had no effect on either the cell number or the curve area (6.07 ± 0.42 vs. 4.97 ± 0.39 and 100% ± 10.72 vs. 148.96% ± 25.59, respectively) (Fig. 8b,c).

### Influence of TRPV1 and TRPM8

The influence of the extracellular vanilloid receptors TRPV1 and TRPM8 on TPMD signaling was analyzed using AMG 9810 and AMTB which are selective antagonist for TRPV1 and TRPM8, respectively, and BCTC, a an inhibitor of both channel subtypes. In primary human corneal stromal cells, BCTC had no effect on either the cell number or normalized curve area when compared to DMEM controls (4.27 ± 0.42 vs. 3.83 ± 0.33 and 100% ± 11.72 vs. 75.97% ± 8.19, respectively) (Fig. 7b,c). AMG 9810 and AMTB also had no effect on either the cell number or normalized curve area (4.16 ± 0.31, 80.1% ± 7.49; 6.43 ± 0.37, 108.17% ± 9.76, respectively) when compared to DMEM controls (5.23 ± 0.41, 100% ± 8.65) (Fig. 7b,c).

In primary mouse corneal stromal cells, BCTC had no effect on the cell number or curve area when compared to DMEM controls (6.77 ± 0.39 vs. 6.90 ± 0.42 and 100% ± 13.46 vs. 127.25% ± 18.59, respectively) (Fig. 8b,c). AMG 9810 reduced the cell number (5.5 ± 0.36, P < 0.05) when compared to DMEM controls (7.1 ± 0.3), but had no effect on the normalized curve area (100% ± 9.38 vs. 71.35% ± 8.63) (Fig. 8b,c). AMTB had no effect on either the cell number or normalized curve area when compared to DMEM controls (7.1 ± 0.3 vs 5.93 ± 0.44; 100% ± 9.38 vs. 95.55% ± 8.97, respectively) (Fig. 8b,c). In human corneal rim tissue, BCTC had no effect on the mean cell distance (180.31 ± 19.27 μm vs. 180.25 ± 18.11 μm).

### Cornea rubbing

**Figure 10:**
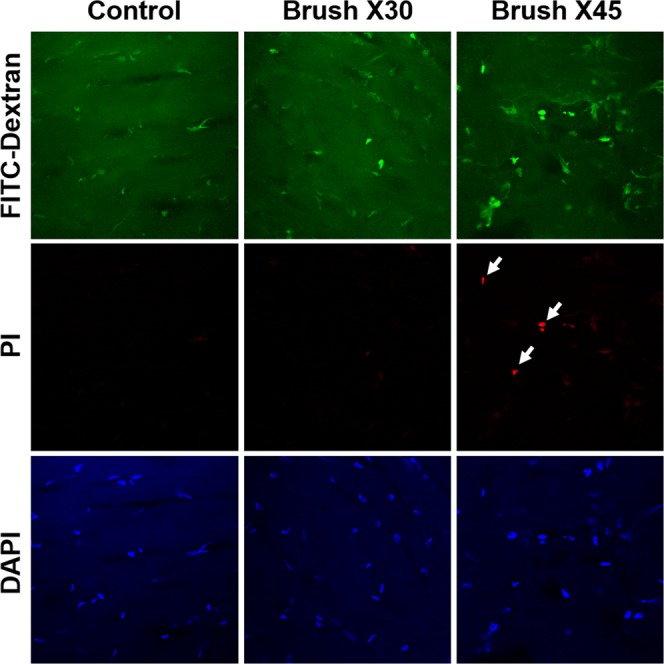
Human corneal rims were rubbed 30 times with a cotton tipped swab to create TPMDs, or 45 times as a positive control for cell death. White arrows point to PI positive dead cells.

**Figure 11:**
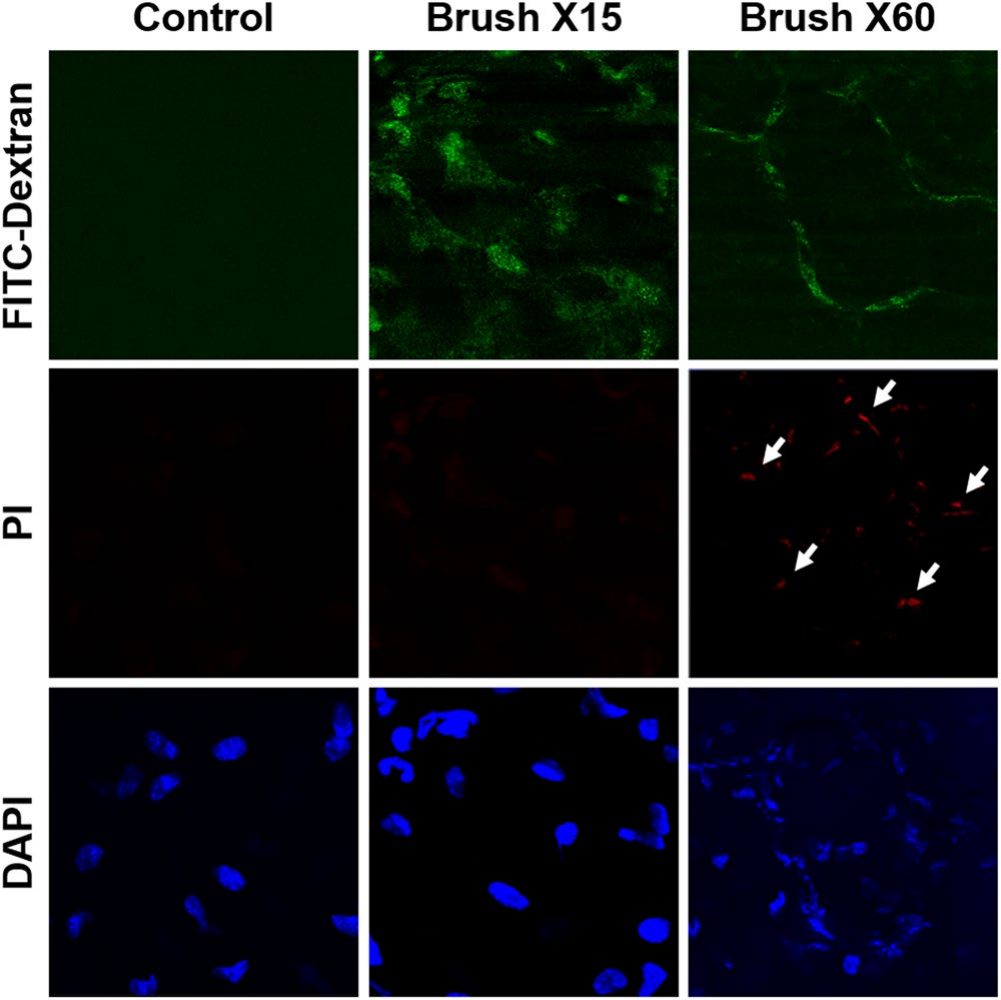
Mouse corneas on intact eyes were rubbed 15 times with a cotton tipped swab to create TPMDs, or 60 times with a hard rounded tip instrument as a positive control for cell damage. White arrows point to PI positive dead cells.

Figures 10 and 11 demonstrate that moderate cornea rubbing in human corneal rims and mouse corneas, respectively, results in dextran-labelled dye entry into living cells, indicative of keratocyte TPMD formation. More aggressive rubbing resulted in TPMDs but also resulted in some cell death. These results confirm that TPMDs do indeed occur in keratocytes living within corneas following mechanical trauma.

## Discussion

The corneal stroma harbors an abundance of quiescent keratocytes that form a cellular network interconnected via gap junctions and numerous intercellular signaling pathways. Keratocyte cell signaling has previously been studied following major disruptions to the different cell types of the cornea or the cornea itself. Transient plasma membrane disruption (TPMD), also referred to as a membrane tear, is a common form of injury to individual cells that can be created by physiological mechanical loading. In the cornea, such physiological loading could theoretically be applied through an act as simple as eye rubbing, or as extreme as corneal wounding during catastrophic injury. The purpose of this study was to determine if TPMD in a single keratocyte can trigger calcium signaling events in neighboring keratocytes.

Calcium waves have previously been reported in corneal epithelial and endothelial cells^20–27^. Trinkaus-Randall’s group have found purinergic signaling to be critical for calcium wave initiation and propagation in corneal epithelial cells, with significant input from corneal nerves^21,25–27^. Importantly, corneal epithelium calcium waves could be initiated by mechanical injury, with 200–400 μm wounds in culture dishes being created using an Argon laser under a confocal microscope^24^. Gap junctions do not appear to be involved in corneal epithelial calcium wave propagation^24^. Corneal epithelial calcium waves appear to support reepithelization after corneal injury^26^.

Corneal endothelial cells show early-and late-responding calcium waves^22^. The early waves can be initiated via mechanical stimulation by deforming the membrane of a single cell with a micropipette, and these waves are propagated through purinergic signaling and gap junctions^20,23^. Late-responding calcium waves in the corneal endothelium occur around an hour after the fast wave, and are related to cell depolarization associated with sodium transport^22^. Endothelial calcium waves appear to play a role in preventing apoptosis^23^. It should be noted that corneal epithelial and endothelial cells are histologically classified as epithelial cell types, while corneal keratocytes are classified as fibroblasts. Corneal epithelium is embryonically derived from surface ectoderm, while keratocytes and endothelium are neural crest-derived. Each cell type has a very different group of ion channels and transporters. The current study is the first to demonstrate TPMD-induced calcium waves in fibroblast-type cells.

TRPV1 and TRPM8 are the only calcium transporters identified in keratocytes to date^18,19^, and no studies have examined cell to cell calcium signaling in keratocytes. In order to accurately analyze TPMD-induced calcium signaling, we chose to examine several parameters in human and mouse cultured stromal cells, and in keratocytes within human corneal rims. In cultured cells we counted the number of cells demonstrating an increase in Ca^++^_i_ following source cell TPMD to represent the general spread of the response. We measured the area under the fluorescence intensity vs. time curve of the responding cells to demonstrate the magnitude and time course of the response. We chose a threshold of ≥50% change in fluorescence (from baseline) to capture definitive changes in Ca^++^_i_. Examining a threshold of 25% resulted in very similar, although not identical results.

Ours is the first study to report and measure TPMD-induced calcium waves in cells residing within any tissue. We were limited in our ability to quantify the response within human corneal stromas, and thus chose to measure the farthest distance between the source cell and a peripheral cell with a clearly changing Ca^++^_i_ within the visual field. A small confocal pinhole size was utilized to create a thin focal plane. The X-Y field of view typically covered the entire area of the calcium wave. The microscope does not have the temporal resolution to do calcium imaging over a large area in the z plane, which would be required to look at 3D dye spread. For this reason, we intentionally limited imaging, to the best of our ability, to the focal plane of the source cell.

It is clear that extracellular calcium plays a significant role in the neighboring cell Ca^++^_i_ response in both human and mouse cells and in resident human keratocytes (Table 1, Figs. 2, 3, 4). The TPMD-induced Ca^++^_i_ response was reduced in all calcium-free medium trials. For the human cell tissue study, thapsigargin treatment resulted in a reduced Ca^++^_i_ response, although the difference was not significant. These experiments were adequately powered to see a significant difference. Taken together with the calcium-free medium response, it appears that the TPMD-induced mouse neighboring cell calcium response is dependent on both intra- and extracellular calcium. The only treatment that affected significantly Ca^++^_i_ in both cultured and stromal human cells was Ca^++^-free K-SFM. This would indicate that the response in human cells is more sensitive to extracellular calcium.

As expected, all trials showed a significant reduction of the Ca^++^_i_ signal following addition of thapsigargin and ryanodine to K-SFM. Interestingly, ryanodine reduced the curve area in MCSC, but increased the corresponding cell number. This may reflect a lower dependence of internal Ca^++^_i_ stores as the source of calcium for TPMD-induced calcium waves. This result may also reflect the more sensitive nature of the curve area measurement versus the cell number measurement. The calcium chelator BAPTA-AM, which served as a positive control for these studies, exerted a powerful role by completely inhibiting calcium signaling in HCSC and MCSC.

In examining the pattern of Ca^++^_i_ increases in cells distributed across the visual field, it was not always clear that responding cells had an obvious physical connection to the source cell or other responding cells. This raised the question about whether or not gap junctions were involved in propagating the response between cells. There is a close relationship between gap junctions and Ca^++^. In some cells, a damage-induced increase of Ca^++^ rapidly closes gap junctions, leading to isolation of healthy cells from damaged neighbors^28^. Alternatively, we have reported that elevated Ca^++^ increases gap junction connectivity between corneal epithelial cells^29^. In astrocytes, mechanically-induced Ca^++^ ion increases and their subsequent propagation to neighboring cells were found to have two distinct patterns: (1) persistent increases were found to propagate rapidly via gap junctions in the proximal region, and (2) transient increases were found to propagate slowly via extracellular ATP in the distal region^11^. It has also been reported that forced expression of gap junction connexins successfully enables gap junction-deficient cell lines to propagate intercellular Ca^++^ waves, stimulating secretion of ATP 5- to 15- fold from the cells^30^. The authors of that study concluded that connexins regulate Ca^++^ signaling by controlling ATP release.

We have previously demonstrated that functional gap junctions exist in human keratocytes within intact corneas^3^, and the presence of Cx43 has been previously reported in cultured human corneal fibroblasts^31^. We confirmed that the primary human keratocytes used in this study contained junctional Cx43 by immunostaining. To determine if gap junctions were propagating the TPMD-induced calcium waves in keratocytes, we used 18α-GA, a gap junction communication inhibitor, to disrupt the gap junction pathway between the TPMD targeted source cell and neighboring cells. In human rim tissue, 18α-GA reduced the calcium signaling maximum distance, but no change was found in HCSC. In MCSC, the curve area was reduced by 18α-GA, while the cell number was unchanged. The difference between human cultured cells and tissue might indicate a more prominent role for gap junctions in relaying the TPMD signal between cells in tissue than in cell culture. Combined with the lack of a cultured human cell response to 18α-GA, the fact that only the curve area was affected in mouse cells indicates that gap junctions likely play only a minor role in communicating the TPMD signal in cultured stromal cells. More detailed studies will need to be conducted to confirm these results.

ATP has been shown to act as a primary signaling molecule directing cell to cell TPMD-induced calcium waves in osteocytes^7^. It was recently reported that the production and release of extracellular vesicles rich in ATP is facilitated by intracellular calcium release in osteocytes^32^. As described above, ATP has also been reported to control peripheral calcium wave propagation in astrocytes. In sea urchin embryos, membrane wounding evoked intracellular calcium ion spikes in neighboring cells, and this phenomenon was mimicked by application of ATP and inhibited by apyrase^33^. ATP efflux is observed in both paracrine and autocrine signaling. Secreted ATP binds to cell surface P2X and/or P2Y purinergic receptors, consequently activating voltage-gated calcium ion channels. Calcium channel activation leads to a calcium ion influx which can, in some cells, activate the PLC-IP3 pathway, producing calcium release from intracellular calcium ion stores^33,34^. Looking at TPMD repair, a recent study in bone cells by Mikolajewicz *et al*.^8^ demonstrated that mechanical stresses induced reversible cell membrane injury that resulted in ATP release to the extracellular space, and that ATP-rich vesicles were critical to patching the cell membrane wound. The vesicle release was Ca2+/PLC/PKC-dependent. To examine the influence of ATP in directing keratocyte calcium waves, keratocytes were treated with apyrase. In the present study, apyrase reduced both the cell number and curve area in HCSC (rim tissue was not tested), but did not affect MCSC. These results further demonstrate a species-dependent signaling response following TPMD, as well as a major difference in the TPMD signaling responses between keratocytes and osteocytes and oocytes.

Transient receptor potential (TRP) channels are non-selective cation channels with variable calcium ion selectivity. The plasma membrane TRPV1 and TRPM8 receptors are highly permeable to calcium, and are the only calcium transporters described to date in keratocytes^18^. To determine if TRPV1 or TRPM8 are involved in the keratocyte TPMD-induced calcium wave response, AMG 9810, a selective antagonist for TRPV1, AMTB, a selective antagonist for TRPM8, and BCTC, an antagonist of both the TRPV1 and TRPM8 channels, were applied to cultured human and mouse stromal cells. In MCSC, AMG 9810 resulted in a small but significant reduction in the cell number measurement, but had no effect on the curve area. No other measurements were affected by the TRP inhibitors. Taken together, this indicates that TRPV1 and TRPM8 do not play a role in the regulation of TPMD-induced keratocyte calcium signaling in HCSC, while TRPV1 may play a small role in MCSC signaling.

Cornea rubbing experiments confirmed that TPMDs do indeed occur in keratocytes following minor mechanical manipulation. It was notable that it took a relatively small amount of rubbing to create TPMD in resident keratocytes versus very serious rubbing to initiate keratocyte cell death in both the human and mouse corneas.

In conclusion, this study is the first to describe TPMD and TPMD-induced calcium signaling in corneal keratocytes. It is also the first to describe ATP-mediated calcium signaling and the involvement of intracellular calcium signaling in keratocytes. It is clear that both intracellular and extracellular calcium are involved in the response, and that gap junctions and ATP play important roles in transmitting the signal from the source cell to neighboring cells and between neighboring cells. TRPV1 and TRPM8 do not appear to be involved in the response. The TPMD-induced signaling pathways are species dependent, and possibly culture versus tissue dependent. Given that keratocyte TPMD is likely a common occurrence following eye rubbing, in dry eye, and following corneal trauma, the TPMD-induced signaling response is likely a normal aspect of corneal physiology and pathophysiology.

## Materials and Methods

### Reagents

Active reagents and the concentrations used were selected from previous TPMD literature^7,35–37^ and from the TRP channel literature^38^. AMG 9810, AMTB, ryanodine, BAPTA-AM, 18-α glycyrrhetinic acid (18α-GA), apyrase and DAPI were purchased from Sigma-Aldrich, St. Louis, MO; thapsigargin, BCTC, and Cal-520-AM dye were purchased from Abcam, Cambridge, United Kingdom, Focus Biomolecules, Plymouth Meeting, PA, and AAT Bioquest, Sunnyvale, CA, respectively. Polyclonal connexin 43 antibody was purchased from Cell Signaling, Danvers, MA; Alexa Fluor 488 was purchased from Fisher, Pittsburgh, PA. Ca^++^-free Keratinocyte-SFM medium (K-SFM) was purchased from ThermoFisher Scientific (Cat. No.: 10725–018), Waltham, MA.

### Isolation and cultivation of primary corneal stromal cells

Primary human corneal stromal cells were isolated and cultured from de-identified human corneal rim tissue^39^ provided by local ophthalmologists. Briefly, fresh human corneal rim tissue was washed with PBS and the epithelial and endothelial layers were removed from the rim under a dissection microscope. The corneal tissue was dissected from the sclera, and corneal tissue was cut into small pieces of about 0.5 × 0.5 mm. After overnight digestion of the corneal tissue in Dulbecco’s modified Eagle’s medium (DMEM) (ThermoFisher Scientific, Waltham, MA) containing collagenase (Sigma, St. Louis, MO) (300 μg/ml, 37 °C), the dissolved materials were collected and centrifuged at 1400 g for 2 min. The resulting pellet was re-suspended in 1.5 ml DMEM, 10% fetal bovine serum (FBS) (HyClone, Pittsburgh, PA), gentamicin (ThermoFisher Scientific, Waltham, MA), insulin-transferrin-selenium (ITS) (ThermoFisher Scientific, Waltham, MA), and cultured on a 35 mm plate (Fisher Scientific, Waltham, MA) in a humidified incubator at 37 °C, 5% CO_2_. Confluent cells were passaged using trypsin/EDTA (Sigma-Aldrich, St. Louis, MO). Use of the donor tissue was in accordance with the Declaration of Helsinki. This study was deemed exempt by the Augusta University IRB committee.

Informed consent was obtained from all donors or their families in accordance with tissue donation protocols.

Isolation and culture of primary mouse corneal stromal cells from 4 week old C57BL/6 mouse were performed as above. The culture medium was replaced with fresh DMEM medium containing 10% FBS every 2 days. Cells of passages 3–6 were used in this study. Cells were routinely screened using PCR for the presence of the corneal stromal cell-specific marker thy-1^39,40^. All protocols for animal use and euthanasia were reviewed and approved by the Augusta University Institutional Animal Care and Use Committee and followed the ARVO Statement for the Use of Animals in Ophthalmic and Vision Research.

### Immunofluorescence staining

Primary human and mouse corneal stromal cells (HCSC and MCSC, respectively) were fixed in cold 4% paraformaldehyde for 10 min followed by washing in phosphate buffer. Cells were incubated in goat serum (10% in phosphate buffer or PBS) for 60 min, washed in 0.1% Triton X-100/PBS, and exposed overnight to anti-connexin 43 (Cx43; 1:200). After rinsing with 0.1% Triton X-100/PBS, cultures were exposed to secondary antibody conjugated to Alexa Fluor 488 (1:1000) for 1 h and washed in 0.1% Triton X-100/PBS. DAPI was added to each slide with mounting medium. Slides were examined using a Zeiss LSM 780 upright laser-scanning confocal microscope equipped with ZEN lite software.

### Laser wounding and calcium signaling in cultured corneal stromal cells

HCSC and MCSC were seeded onto 35 mm plates 24 h prior to experiments and stained with the calcium indicator Cal-520-AM (0.25 μM) as described^41^. Various treatments were introduced 1 hour before the membrane disruption procedure: thapsigargin (1 μM; sarco/endoplasmic reticulum Ca^2+^ ATPase inhibitor); ryanodine (50 uM; sarco/endoplasmic reticulum Ca^++^ release blocker); BAPA-AM (50 uM; membrane permeable calcium chelator); 18α-GA (30 μM; gap junction inhibitor); apyrase (30 min) (10 units/ml; catalyzes hydrolysis of ATP); BCTC (10 μM; TRPV1 and TRPM8 receptor antagonist); AMG 9810 (30 min) (10 uM; selective TRPV1 antagonist); AMTB (30 min) (10 uM; selective TRPM8 antagonist). Cells in DMEM medium were used as controls. Ca^++^-free Keratinocyte-SFM medium (K-SFM; ThermoFisher Scientific, Cat. No.: 10725–018) was used as the Ca^++^-free medium with K-SFM plus 1 mM Ca^++^ used as the Ca^++^ control. Cells were in the calcium-free KSFM for 15 min. minutes before the experiment started, and experiments typically took 30 min. For cell culture experiments, the same culture passage was used to compare control and experimental groups.

Single TPMDs were produced in Cal-520-AM pre-stained corneal stromal cells using a Zeiss 780 upright multi-photon microscope, using a procedure slightly modified from that described previously by one of the authors (MML)^9^. Briefly, an 820 nm laser was used to produce a TPMD in a single targeted cell using the microscope’s single-time bleaching setting. A circular 9 μm diameter region was targeted on the cell and a single 100% power pulse created the damage. Cal-520-AM fluorescence was visualized and recorded for 50 seconds under a 20x objective (Zeiss W Plan-Apochromat N.A. = 1, working distance = 2.4 mm) using a 488 nm laser. A series of 50 scans was performed at a scan speed of 1 scan/second.

5–10 source cells per culture plate in isolated fields of view were separately targeted for TPMD. The fluorescence intensity of other cells within that field of view that had a notable increase in fluorescent intensity was measured using ZEN 2.1 software by manually tracing those cells and measuring the fluorescent intensity of each cell over the time course of the trial. For the final analysis, only cells that increased their baseline fluorescent intensity by a minimum of 50% were considered positive responders. Both the number of positively responding neighboring cells (cell number) and the area under the fluorescence intensity versus time curve (curve area) of responding cells, which takes into account both the change in Ca^++^ concentration along with the duration of that change, were measured. Curve area values were normalized to control for comparisons. The baseline-corrected curve area was calculated using the formula Area = ∑ (I_n+1_ + I_n_)/2*(T_n+1_ − T_n_), where, I: fluorescent intensity, T: time. Each experiment included at least 3 plates.

### Laser wounding and calcium signaling in fresh human corneal tissue

For all trials except the K-SFM + thapsigargin trial, human keratoplasty corneal rims were divided in half; one half was used as the DMEM control and the other half used for treatment. The rims treated with K-SFM + thapsigargin were compared to unmatched rims in K-SFM + 1 mM calcium. Cal-520-AM staining and drug treatments were performed as described above. After washing with PBS, the corneal tissue was pinned onto a silicone pad within a 35 mm plate with the endothelium surface facing up. The 820 nm laser was used to produce a TPMD in a single targeted cell using the microscope’s multiple-time bleaching setting. The bleaching region was set as above with 100% power, with repeated bleaching after every 3 scans. The confocal pinhole setting was set to 1.26 Airy units, corresponding to a 1.80 μm optical section, to minimize out of focal plane cell imaging. All scan settings were set as above.

Image resolution limitations prevented us from using the same measurements used in the cultured cell studies. Instead, for these experiments, the neighboring cell farthest from the source cell with a notable change in fluorescence within the visual field was noted, and the distance from the source cell to that cell to was measured (cell distance). The mean maximum distance from approximately 10 targeted source cells per rim section was calculated and used for statistical analysis. The mean maximum cell distance was compared between DMEM control groups and treatment groups. Assays were repeated in at least 3 rims per experimental group.

### TPMD after cornea rubbing

TPMDs have never been shown in cells residing within their native tissue. To determine if keratocyte TPMDs can occur as a result of the equivalent of eye rubbing, mice were sacrificed and a mixture of fluorescein-labelled dextran (10 kDa, 0.5 mg/ml) and propidium iodide (PI, Sigma-Aldrich, St. Louis, MO) (0.5 mg/ml) was injected into the anterior chamber. Corneas were then rubbed 30 times with a cotton-tipped swab using moderate pressure to mimic moderate and heavy eye rubbing^42,43^. For positive controls of cell damage/death, corneas were rubbed 60 times with the rounded bottom end of a scalpel. We determined that this relatively extreme method was required to fatally damage cells using this method. Corneas were then isolated, fixed in 4% paraformaldehyde (Electron Microscopy Sciences, Hatfield, PA), stained with DAPI (Santa Cruz Biotechnology, Dallas, TX), and mounted on a microscope slide for multiphoton fluorescence imaging. FITC-dextran can only label cells that have a membrane disruption, while PI stains the nuclei of dead cells, providing a view of which cells had FITC-dextran staining as a result of TPMDs, and which as a result of plasma membrane disruption following cell death. Human corneal rim tissue was subjected to rubbing with a cotton-tipped swab. The same FITC-dextran/PI mixture was added to DMEM in a cell culture dish, the rim was rubbed 0, 30 and 45 times, and the cornea was fixed, stained with DAPI and imaged as above. This protocol was modeled after the mechanical injury (glass beads) protocol described in^7^.

### Statistical analysis

All data are provided as the mean ± SE of at least three experiments. The difference between the DMEM control groups and treatment groups was compared using the Student’s t-test (GraphPad Prism 7.0), where P < 0.05 was considered statistically significant.

## Supplementary information

Supplementary Video Legends

Supplementary Video S1.

Supplementary Video S2.

Supplementary Video S3.

Supplementary Video S4. 

Supplementary Video S5.

Supplementary Video S6.

Supplementary Video S7. 

Supplementary Video S8.

Supplementary Video S9.

Supplementary Video S10.

Supplementary Video S11.

Supplementary Video S12.

Supplementary Video S13.

Supplementary Video S14.

Supplementary Video S15.

Supplementary Video S16.

Supplementary Video S17.

Supplementary Video S18.

Supplementary Video S19.

Supplementary Video S20.

Supplementary Video S21.

Supplementary Video S22.

Supplementary Video S23.

Supplementary Video S24.

Supplementary Video S25.

Supplementary Video S26.

Supplementary Video S27.

Supplementary Video S28.

## Data Availability

The datasets generated during in this study are available from the corresponding author on reasonable request.
