# Supplementary material for: Transient Cell Membrane Disruptions induce Calcium Waves in Corneal Keratocytes
Source: Sci Rep. 2020 Feb 18;10:2840. doi: 10.1038/s41598-020-59570-7 (PMC7029045; doi:10.1038/s41598-020-59570-7)
Supplement: Supplementary file 1 — Supplementary Video Legends [file 41598_2020_59570_MOESM1_ESM.docx]

**Supplementary Materials**

**Transient Cell Membrane Disruptions induce Calcium Waves in Corneal Keratocytes**

Zhong Chen, Xiaowen Lu, Meghan E. McGee-Lawrence, Mitchell A. Watsky

**Supplementary Figure Legends**

**Supplementary Video S1**. Primary Human Corneal Stromal Cell Calcium Wave Video, K-SFM + Calcium, 20X objective. Calcium wave spread among primary human stromal cells. Still photos in Fig. 2a captured from this video. Circle highlights the TPMD target on the source cell.

**Supplementary Video S2**. Primary Human Corneal Stromal Cell Calcium Wave Video, K-SFM, 20X objective. Calcium wave spread among primary human stromal cells. Still photos in Fig. 2a captured from this video. Circle highlights the TPMD target on the source cell.

**Supplementary Video S3**. Primary Human Corneal Stromal Cell Calcium Wave Video, K-SFM + Thapsigargin, 20X objective. Calcium wave spread among primary human stromal cells. Still photos in Fig. 2a captured from this video. Circle highlights the TPMD target on the source cell.

**Supplementary Video S4**. Primary Human Corneal Stromal Cell Calcium Wave Video, K-SFM + Ryanodine, 20X objective. Calcium wave spread among primary human stromal cells. Still photos in Fig. 2a captured from this video. Circle highlights the TPMD target on the source cell.

**Supplementary Video S5**. Primary Human Corneal Stromal Cell Calcium Wave Video, K-SFM + BAPTA-AM, 20X objective. Calcium wave spread among primary human stromal cells. Still photos in Fig. 2a captured from this video. Circle highlights the TPMD target on the source cell.

**Supplementary Video S6**. Primary Mouse Corneal Stromal Cell Calcium Wave Video, K-SFM + Calcium, 20X objective. Calcium wave spread among primary mouse stromal cells. Still photos in Fig. 3a captured from this video. Circle highlights the TPMD target on the source cell.

**Supplementary Video S7**. Primary Mouse Corneal Stromal Cell Calcium Wave Video, K-SFM, 20X objective. Calcium wave spread among primary mouse stromal cells. Still photos in Fig. 3a captured from this video. Circle highlights the TPMD target on the source cell.

**Supplementary Video S8.** Primary Mouse Corneal Stromal Cell Calcium Wave Video, K-SFM + Thapsigargin, 20X objective. Calcium wave spread among primary mouse stromal cells. Still photos in Fig. 3a captured from this video. Circle highlights the TPMD target on the source cell.

**Supplementary Video S9**. Primary Mouse Corneal Stromal Cell Calcium Wave Video, K-SFM + Ryanodine, 20X objective. Calcium wave spread among primary mouse stromal cells. Still photos in Fig. 3a captured from this video. Circle highlights the TPMD target on the source cell.

**Supplementary Video S10**. Primary Mouse Corneal Stromal Cell Calcium Wave Video, K-SFM + BAPTA-AM, 20X objective. Calcium wave spread among primary mouse stromal cells. Still photos in Fig. 3a captured from this video. Circle highlights the TPMD target on the source cell.

**Supplementary Video S11**. Human Corneal Rim Tissue Calcium Wave Video, K-SFM, 20X objective. Calcium wave spread among keratocytes within a human corneal rim. Still photos in Fig. 4a captured from this video. A red arrow points to the source cell and a white circle indicates the farthest affected cell.

**Supplementary Video S12**. Human Corneal Rim Tissue Calcium Wave Video, K-SFM + Calcium, 20X objective. Calcium wave spread among keratocytes within a human corneal rim. Still photos in Fig. 4a captured from this video. A red arrow points to the source cell and a white circle indicates the farthest affected cell.

**Supplementary Video S13**. Human Corneal Rim Tissue Calcium Wave Video, DMEM, 20X objective. Calcium wave spread among keratocytes within a human corneal rim. Still photos in Fig. 5a captured from this video. A red arrow points to the source cell and a white circle indicates the farthest affected cell.

**Supplementary Video S14**. Human Corneal Rim Tissue Calcium Wave Video, DMEM + Thapsigargin, 20X objective. Calcium wave spread among keratocytes within a human corneal rim. Still photos in Fig. 5a captured from this video. A red arrow points to the source cell and a white circle indicates the farthest affected cell.

**Supplementary Video S15**. Primary Human Corneal Stromal Cell Calcium Wave Video, DMEM,

20X objective. Calcium wave spread among primary human stromal cells. Still photos in Fig. 7a captured from this video. Circle highlights the TPMD target on the source cell.

**Supplementary Video S16**. Primary Human Corneal Stromal Cell Calcium Wave Video, DMEM + BCTC, 20X objective. Calcium wave spread among primary human stromal cells. Still photos in Fig. 7a captured from this video. Circle highlights the TPMD target on the source cell.

**Supplementary Video S17**. Primary Human Corneal Stromal Cell Calcium Wave Video, DMEM + AMG 9810, 20X objective. Calcium wave spread among primary human stromal cells. Still photos in Fig. 7a captured from this video. Circle highlights the TPMD target on the source cell.

**Supplementary Video S18**. Primary Human Corneal Stromal Cell Calcium Wave Video, DMEM + AMTB, 20X objective. Calcium wave spread among primary human stromal cells. Still photos in Fig. 7a captured from this video. Circle highlights the TPMD target on the source cell.

**Supplementary Video S19**. Primary Human Corneal Stromal Cell Calcium Wave Video, DMEM + 18α-GA, 20X objective. Calcium wave spread among primary human stromal cells. Still photos in Fig. 7a captured from this video. Circle highlights the TPMD target on the source cell.

**Supplementary Video S20**. Primary Human Corneal Stromal Cell Calcium Wave Video, DMEM + Apyrase, 20X objective. Calcium wave spread among primary human stromal cells. Still photos in Fig. 7a captured from this video. Circle highlights the TPMD target on the source cell.

**Supplementary Video S21**. Primary Mouse Corneal Stromal Cell Calcium Wave Video, DMEM,

20X objective. Calcium wave spread among primary mouse stromal cells. Still photos in Fig. 8a captured from this video. Circle highlights the TPMD target on the source cell.

**Supplementary Video S22**. Primary Mouse Corneal Stromal Cell Calcium Wave Video, DMEM + BCTC, 20X objective. Calcium wave spread among primary mouse stromal cells. Still photos in Fig. 8a captured from this video. Circle highlights the TPMD target on the source cell.

**Supplementary Video S23**. Primary Mouse Corneal Stromal Cell Calcium Wave Video, DMEM + AMG 9810, 20X objective. Calcium wave spread among primary mouse stromal cells. Still photos in Fig. 8a captured from this video. Circle highlights the TPMD target on the source cell.

**Supplementary Video S24**. Primary Mouse Corneal Stromal Cell Calcium Wave Video, DMEM + AMTB, 20X objective. Calcium wave spread among primary mouse stromal cells. Still photos in Fig. 8a captured from this video. Circle highlights the TPMD target on the source cell.

**Supplementary Video S25**. Primary Mouse Corneal Stromal Cell Calcium Wave Video, DMEM + 18α-GA, 20X objective. Calcium wave spread among primary mouse stromal cells. Still photos in Fig. 8a captured from this video. Circle highlights the TPMD target on the source cell.

**Supplementary Video S26**. Primary Mouse Corneal Stromal Cell Calcium Wave Video, DMEM + Apyrase, 20X objective. Calcium wave spread among primary mouse stromal cells. Still photos in Fig. 8a captured from this video. Circle highlights the TPMD target on the source cell.

**Supplementary Video S27**. Human Corneal Rim Tissue Calcium Wave Video, DMEM, 20X objective. Calcium wave spread among keratocytes within a human corneal rim. Still photos in Fig. 9a captured from this video. A red arrow points to the source cell and a white circle indicates the farthest affected cell.

**Supplementary Video S28**. Human Corneal Rim Tissue Calcium Wave Video, DMEM + 18α-GA, 20X objective. Calcium wave spread among keratocytes within a human corneal rim. Still photos in Fig. 9a captured from this video. A red arrow points to the source cell and a white circle indicates the farthest affected cell.
